# Supplementary material for: Repeated measures of decaying wood reveal the success and influence of fungal wood endophytes
Source: mSystems. 2025 Aug 15;10(9):e00382-25. doi: 10.1128/msystems.00382-25 (PMC12455916; doi:10.1128/msystems.00382-25)
Supplement: Supplemental Figures — Fig. S1 to S16. [file msystems.00382-25-s0001.pdf]

**Supplementary Figures: Fig. S1 to Fig. S16**

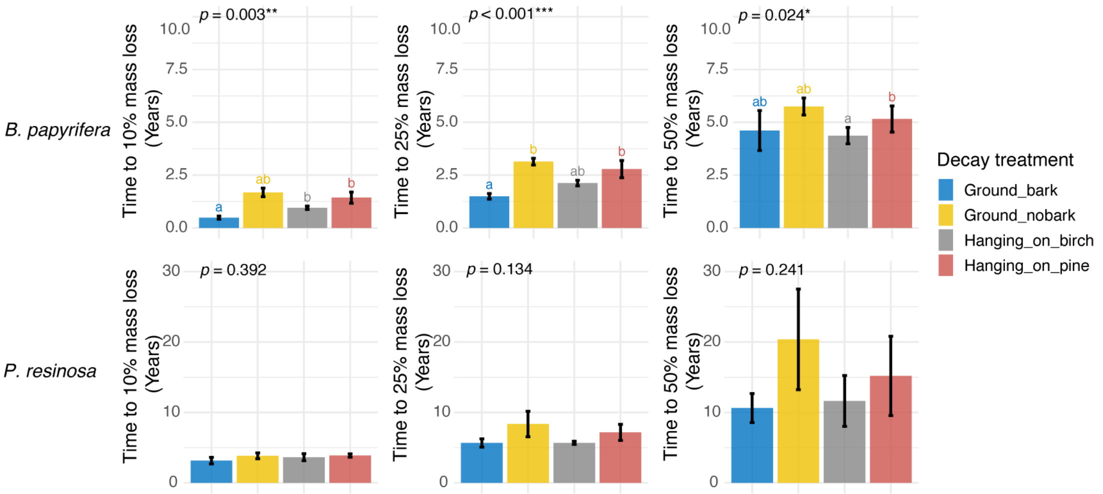


**Fig. S1 Time to 10% mass loss, 25% mass loss, and 50% mass loss of *B. papyrifera* and *P. resinosa* for different treatments.** The "years remaining" residence times for each tree log were assessed by a Weibull residence. The *P* values indicate the significance test of treatment using the Friedman test. The significance is shown: $*P < 0.05$, $**P < 0.01$, $***P < 0.001$. The different lowercase letters indicate the significance of the pairwise paired Wilcoxon test.

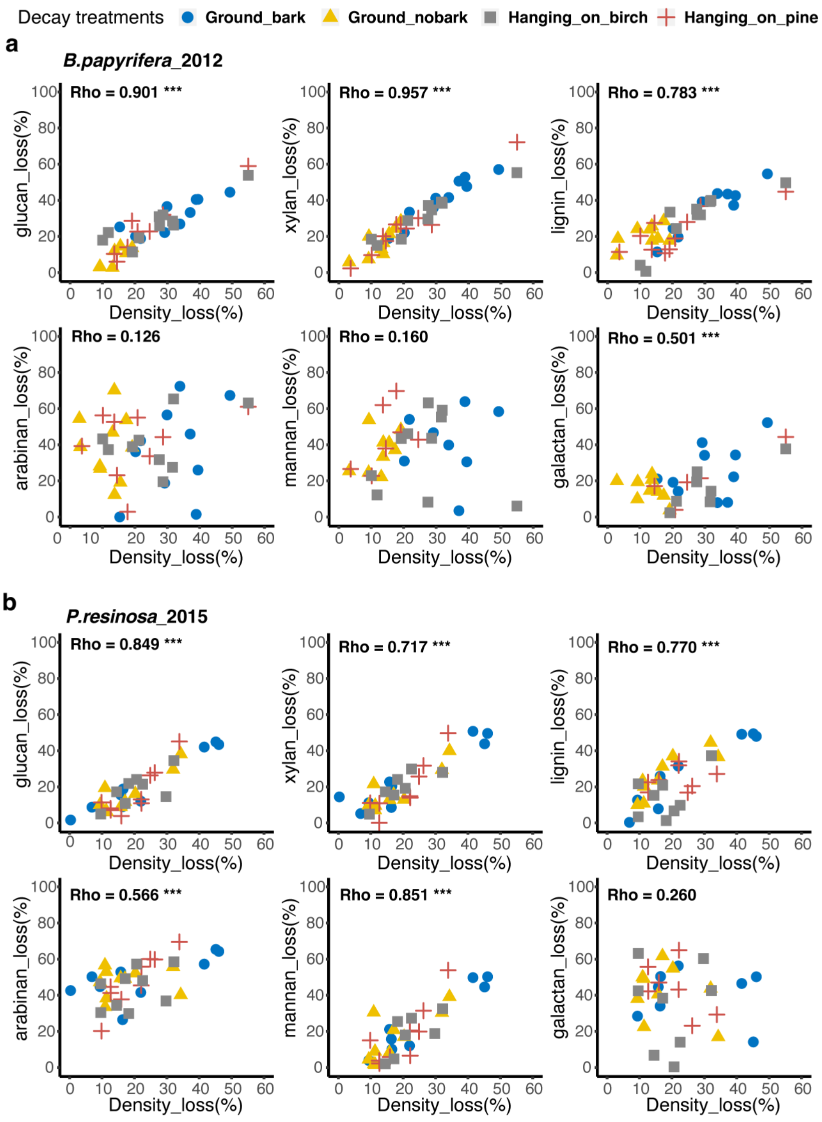


**Fig. S2 The main carbohydrate loss was positively correlated with density loss.** Spearman's correlations between carbohydrate loss and density loss for (**a**) *B. papyrifera* after 2 years of decay and (**b**) *P. resinosa* after 5 years. Spearman's rank correlation rho is shown with the significance test: $*P < 0.05$, $**P < 0.01$, $***P < 0.001$.

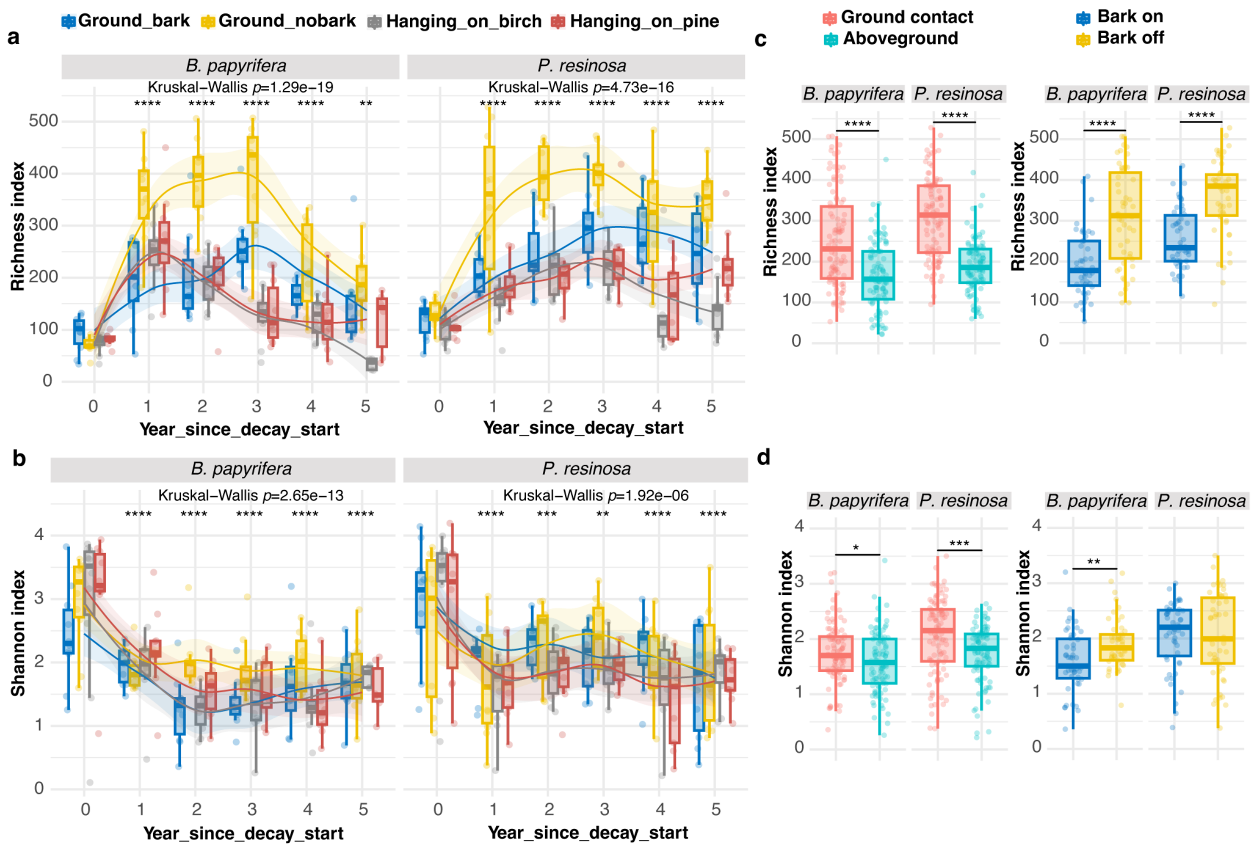


**Fig. S3 Alpha diversity of fungi in the decay process of *B. papyrifera* and *P. resinosa* for different treatments.** (**a**) Species richness and (**b**) Shannon index of the fungal community over time. The * indicates significant differences between each time point and the reference group of sound wood samples (Time 0) for each tree host independently, according to Wilcoxon tests. Aboveground effect and bark effect on (**c**) fungal richness and (**d**) Shannon index in the decay process of *B. papyrifera* and *P. resinosa*. The * indicated significant differences between certain treatment groups for each tree host independently according to the Wilcoxon tests. Significant test: *$P < 0.05$, **$P < 0.01$, ***$P < 0.001$, ****$P < 0.0001$.

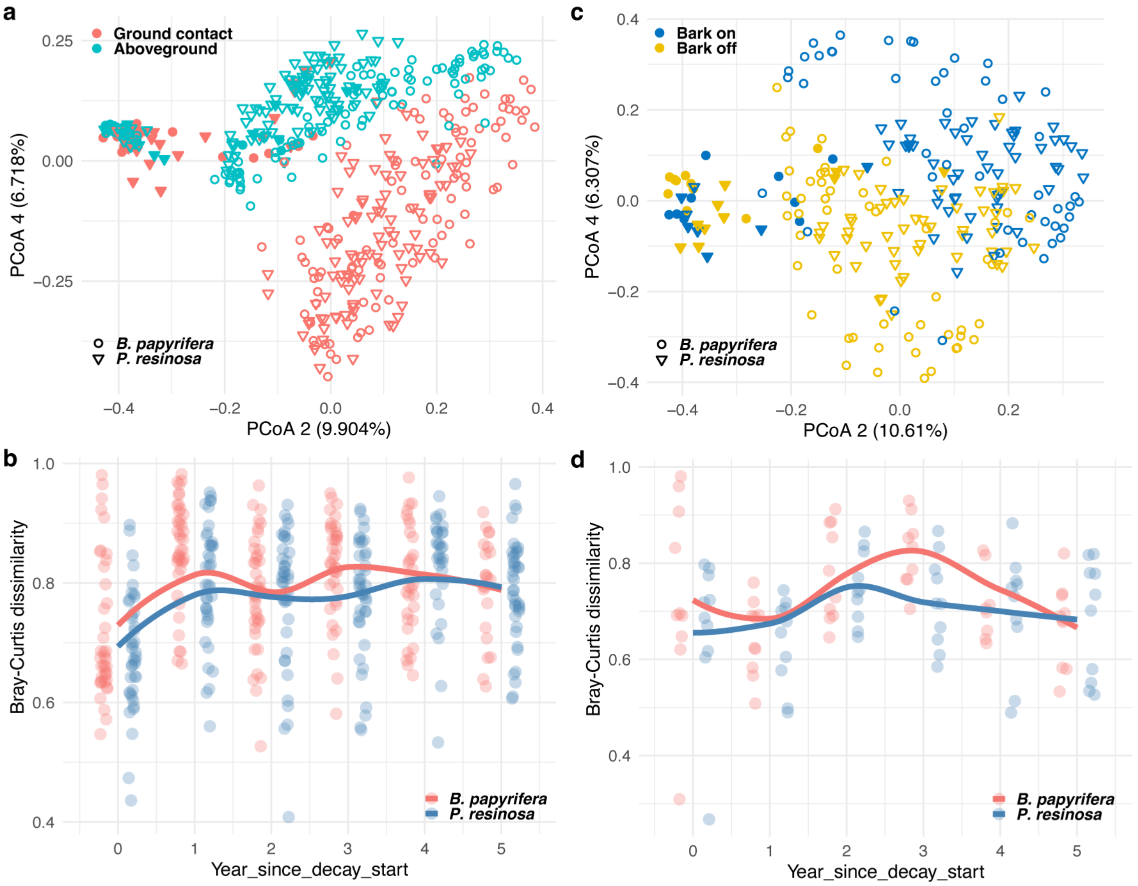


**Fig. S4 The treatment of aboveground or bark removal caused fungal community composition differences.** Principal coordinates analysis (PCoA) of Bray-Curtis dissimilarity of fungal communities between (**a**) ground contact/aboveground samples and (**c**) between bark on/bark off samples. Samples are shaped by tree host and colored by treatments. The sound and decayed wood samples are represented by filled and open symbols, respectively. Pairwise distances (**b**) between contact/aboveground samples and (**d**) between bark on/bark off samples within each decay time and each tree host.

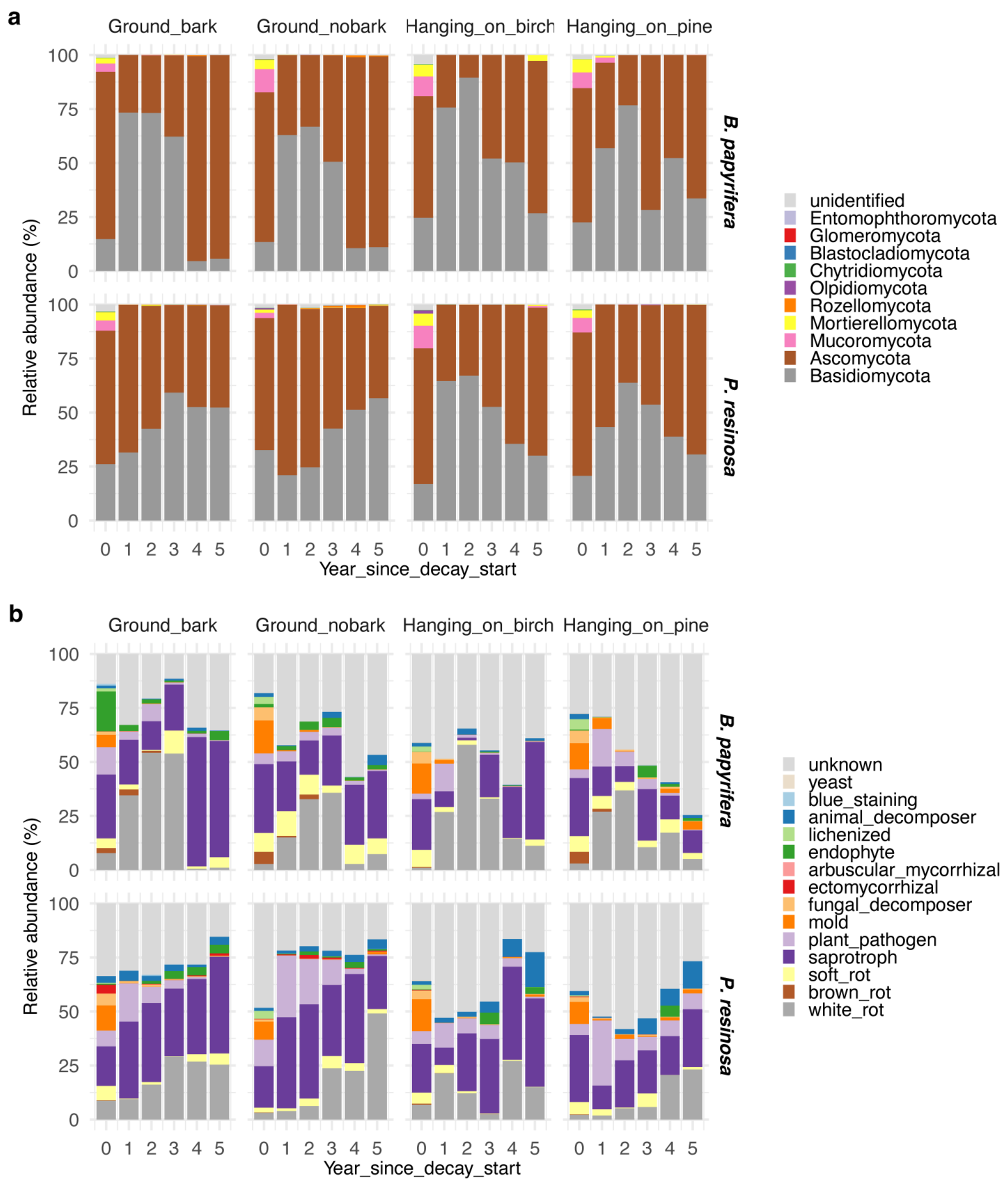


**Fig. S5 Succession of fungal communities *B. papyrifera* and *P. resinosa*.** The fungal composition at (**a**) the phylum level and (**b**) their ecological group. The ecological group was assigned based on the genus level using the FungalTrait database.

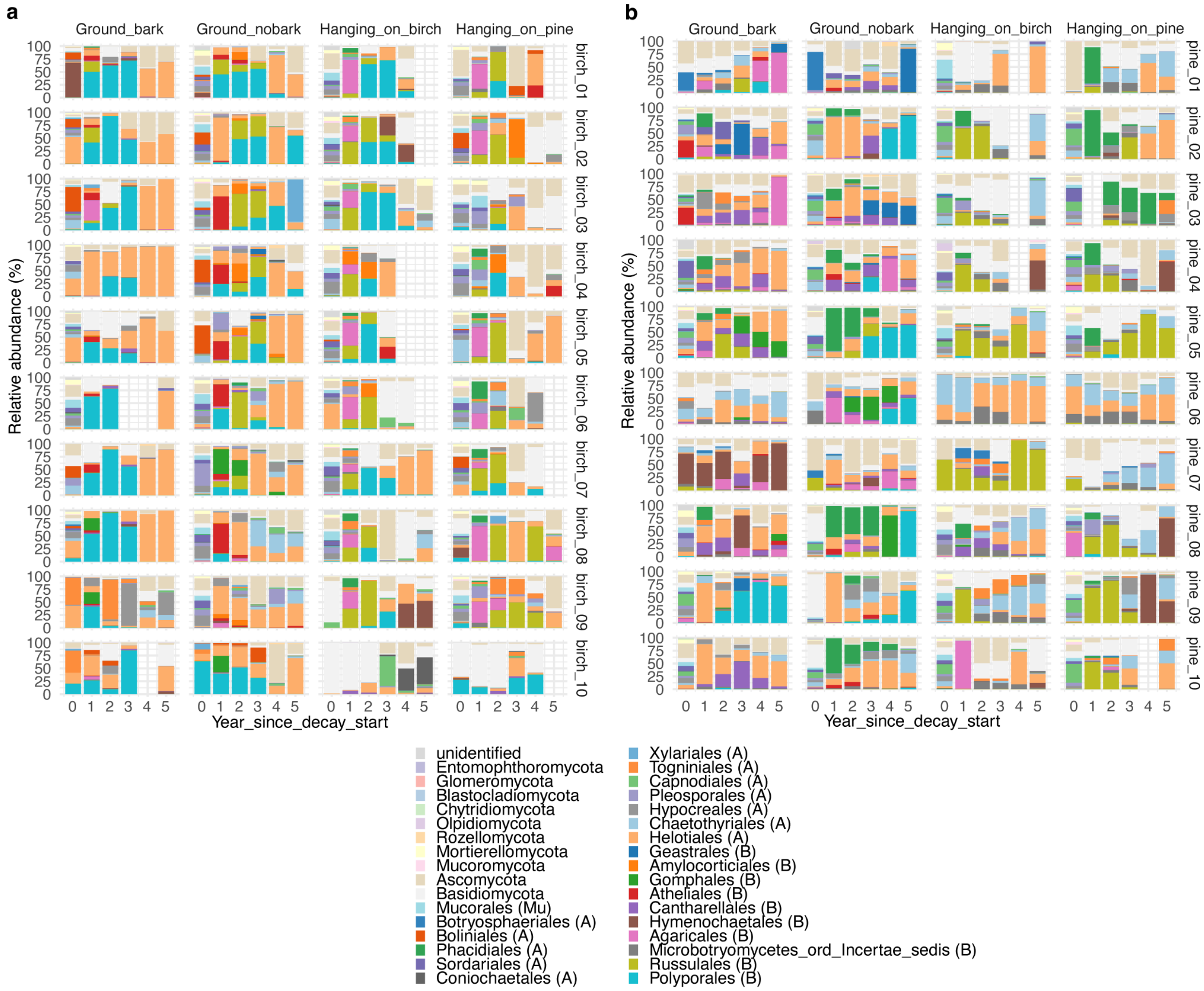


**Fig. S6 Relative abundances of fungal orders in the decay process of the individual log of *B. papyrifera* (a) and *P. resinosa* (b).** Only orders with at least 7% relative abundance in one of the treatments were specified, and all others were classified to the phylum level. All the taxa not assigned to the level of order were also classified to the phylum level. Abbreviations: A: Ascomycota; B: Basidiomycota; Mu: Mucoromycota.

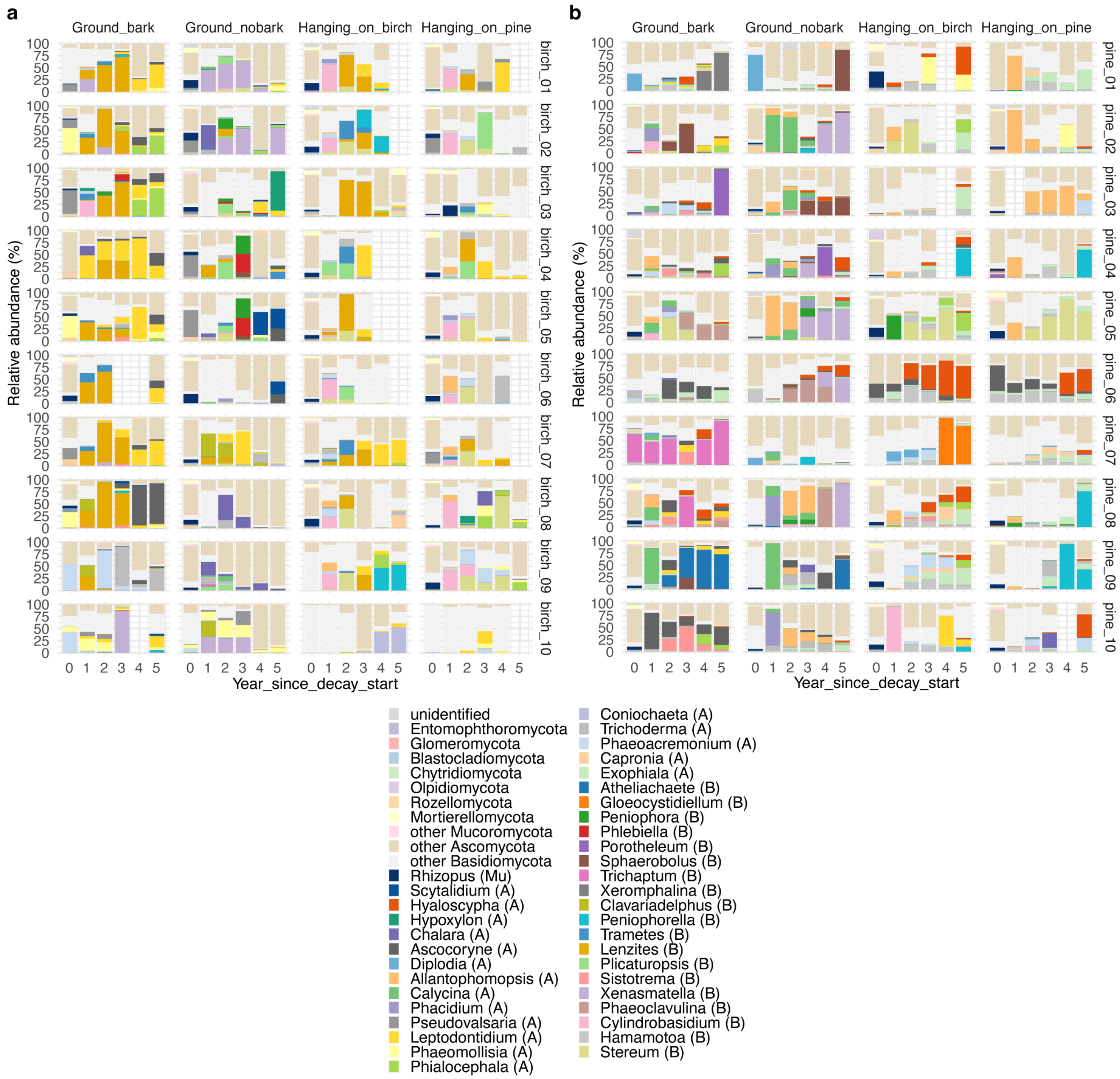


**Fig. S7 Relative abundances of fungal genera in the decay process of the individual log of *B. papyrifera* (a) and *P. resinosa* (b).** Only genera with at least 7% relative abundance in one of the treatments were specified, and all others were classified to the phylum level. All the taxa that were not assigned to the level of genus were also classified to the phylum level. Abbreviations: A: Ascomycota; B: Basidiomycota; Mu: Mucoromycota.

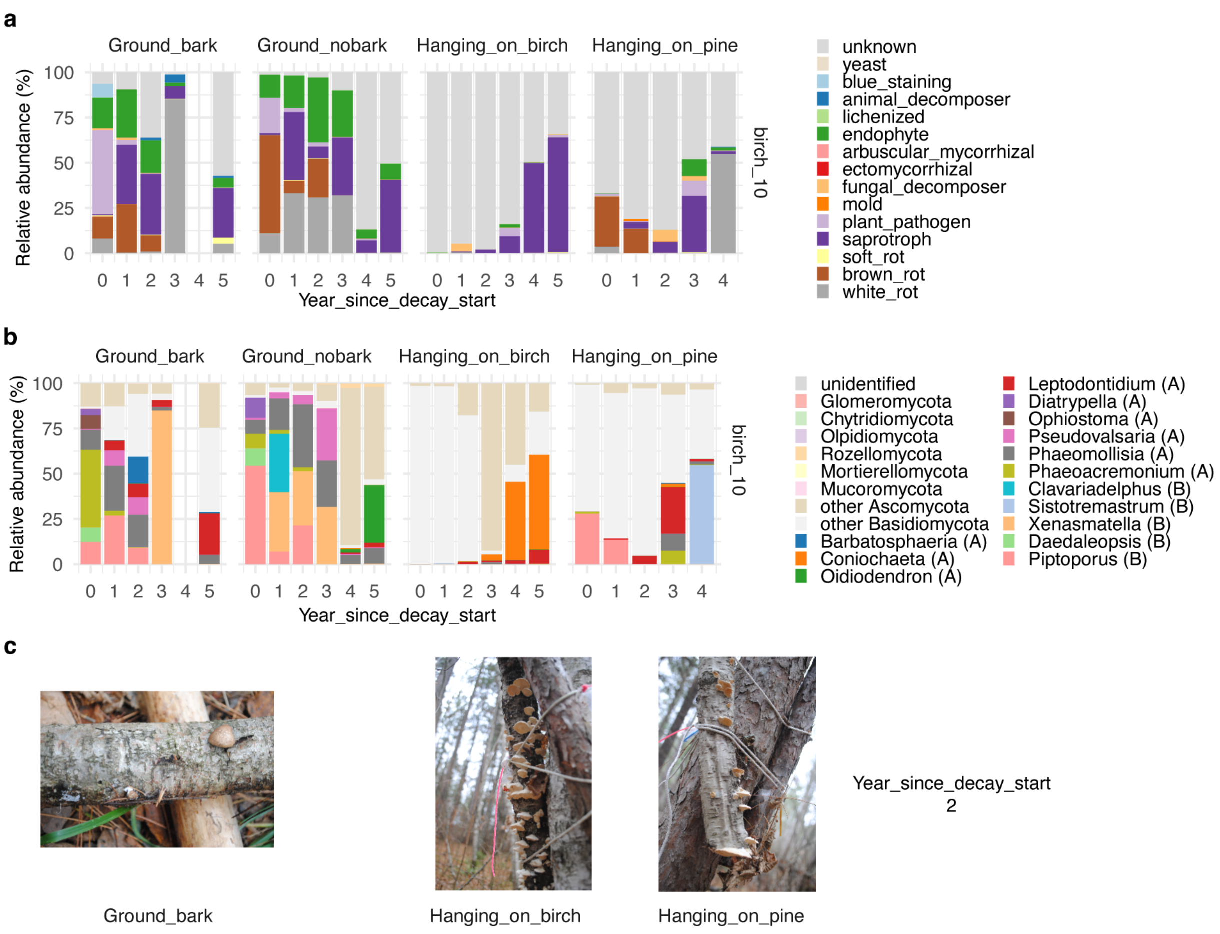


**Fig. S8 Fungal community succession over the decaying of an individual log of *B. papyrifera* (birch_10).** Relative abundance of fungi in functional ecology group (**a**) and at the genus level (**b**). (**c**) Fruiting pictures of individual logs after 2 years of decaying. Only genera with at least 7% relative abundance in one of the treatments were specified, and all others were classified to the phylum level. All the taxa that were not assigned to the level of genus were also classified to the phylum level.

Abbreviations: A: Ascomycota; B: Basidiomycota.

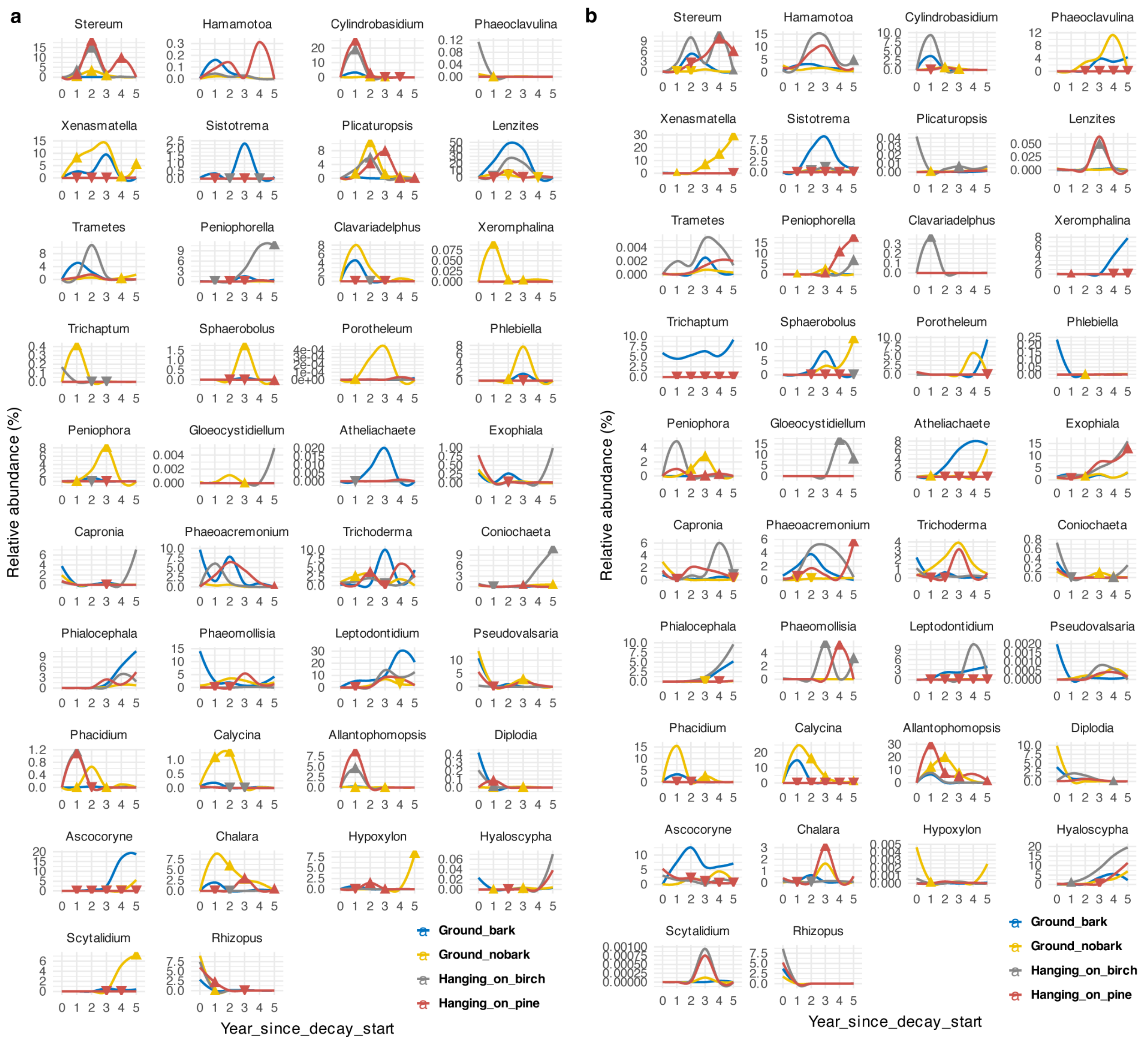


**Fig. S9 Comparison of dominant fungi in different treatments in *B. papyrifera* (a) and *P. resinosa* (b).** The ground contact bark on samples was used as a control and compared to each of the other three treatments according to "edgeR" package in R. The upward triangle indicated significantly enriched, and the downward triangle indicated significantly depleted taxa for each tree host independently. The genus chosen here was the same as Figure 4, with at least 7% relative abundance in one of the treatments.

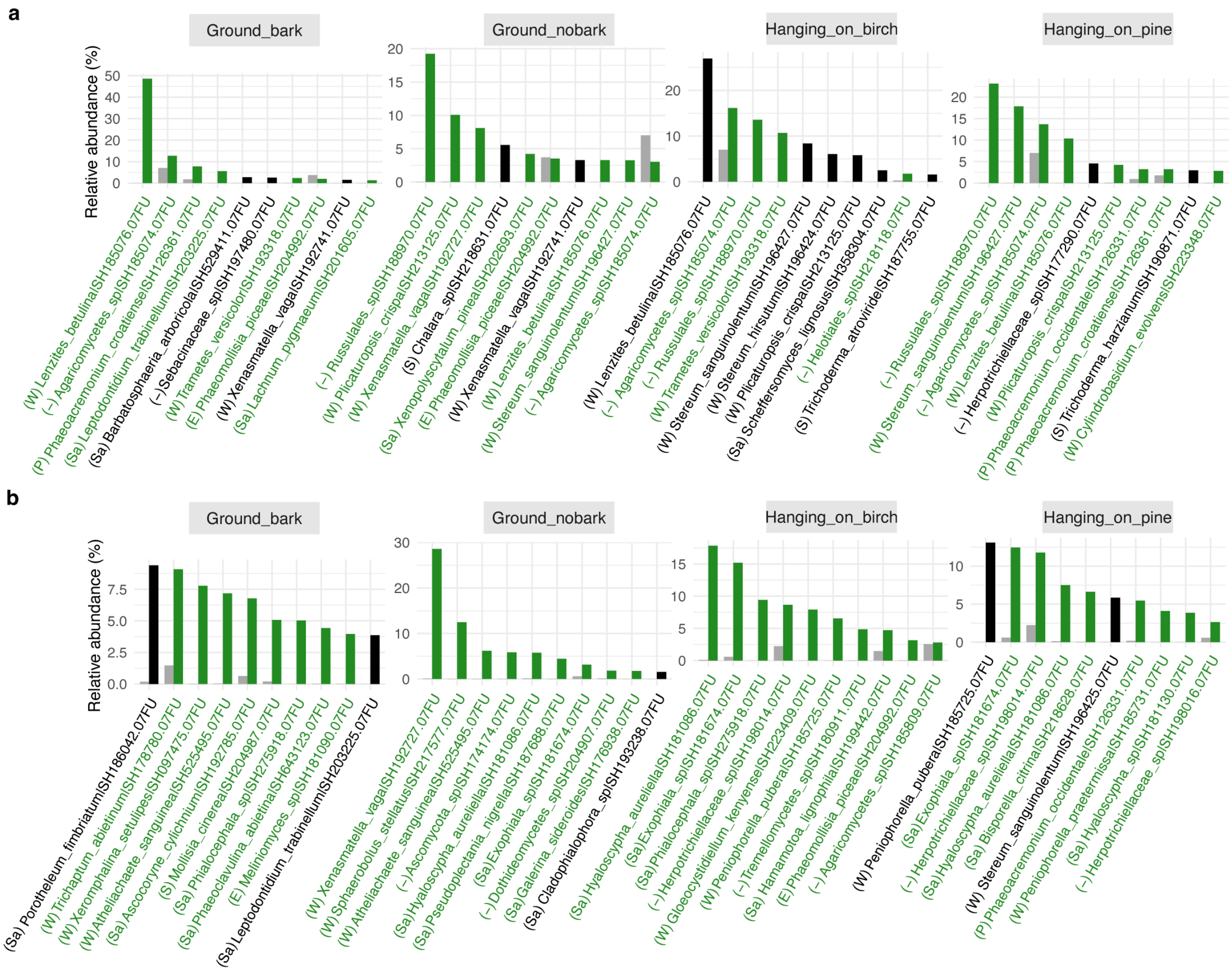


**Fig. S10 The read count percentage of endophytes and external colonizers in the top 10 abundant fungi for birch at year 2 (a) and pine at year 5 (b).** The green bar represented endophytes, while the back bar represented external colonizers. The grey bar showed their relative abundance at time 0. The letters in the parathesis show the ecological group as assigned based on genus level using the FungalTrait database. Abbreviations: Sa, Saprotroph; W, White rot; E, Ectomycorrhizal fungi; P, Plant pathogen; -, Unknown.

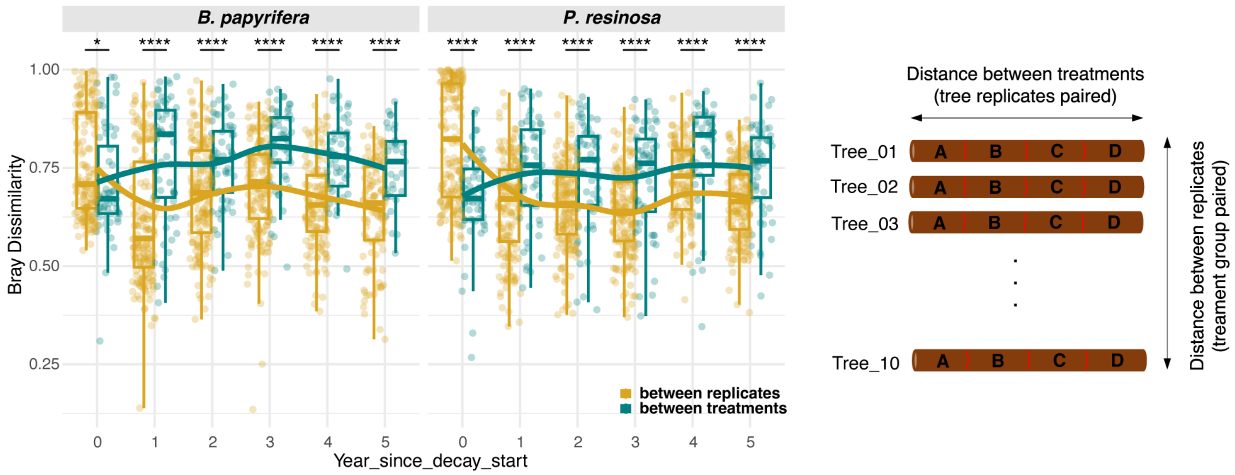


**Fig. S11**. **Comparison of pairwise Bray-Curtis distances of fungal communities between each treatment and between biological replicates within each sampling time and each tree host.** As shown in the illustration, the Bary-Curtis distance between treatments was paired on a tree-by-tree basis, while the distance between replicates was paired on a treatment-by-treatment basis, again benefiting from a repeated measures design. Significant test by the Wilcoxon test: *$P < 0.05$, **$P < 0.01$, ***$P < 0.001$, ****$P < 0.0001$.

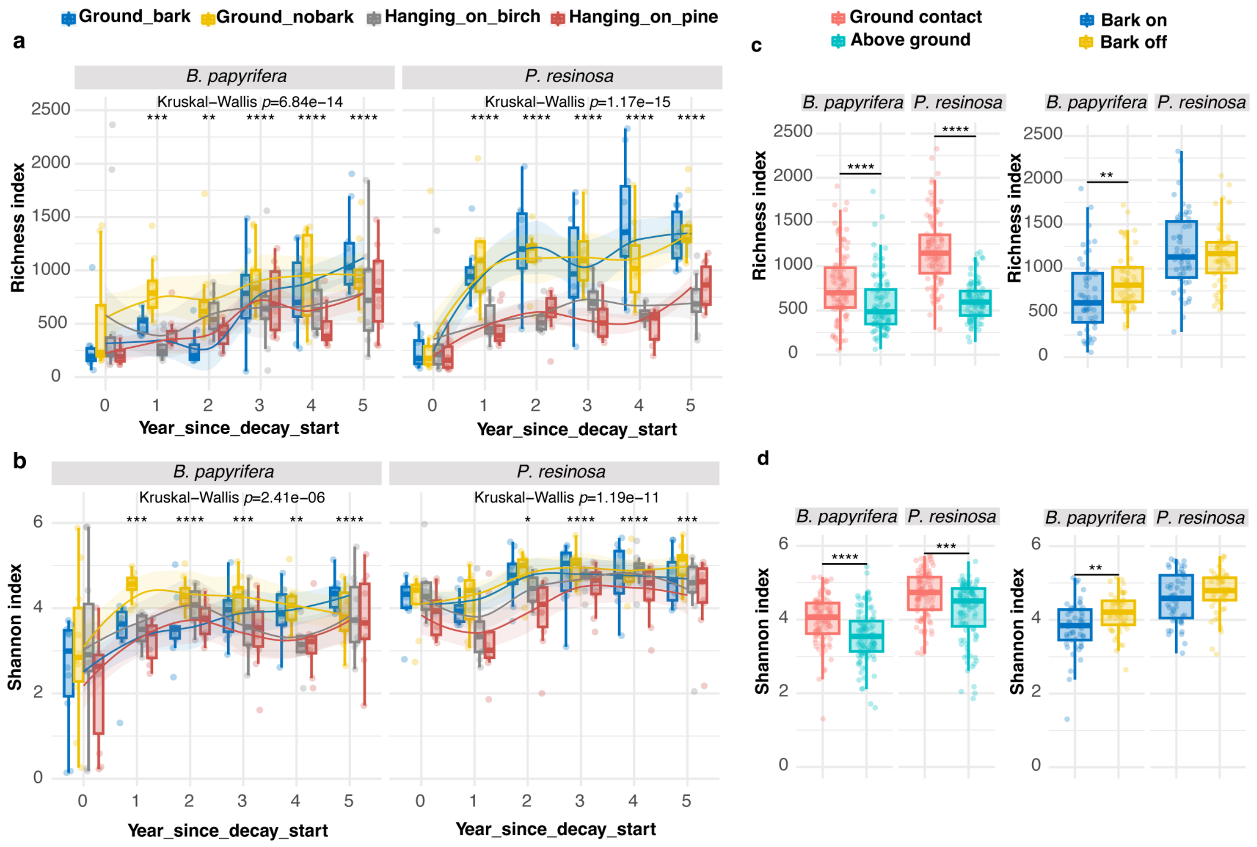


**Fig. S12 Alpha diversity of bacteria in the decay process of *B. papyrifera* and *P. resinosa* for different treatments.** (**a**) Species richness and (**b**) Shannon index of bacterial community over time. The * indicated significant differences between each decay time and the reference group of sound wood samples (Time 0) for each species independently, according to Wilcoxon tests. Aboveground effect and bark effect on (**c**) bacterial richness and (**d**) Shannon diversity in the decay process of *B. papyrifera* and *P. resinosa*. The * indicated significant differences between certain treatment groups for each tree host independently according to the Wilcoxon tests. Significant test: *$P < 0.05$, **$P < 0.01$, ***$P < 0.001$, ****$P < 0.0001$.

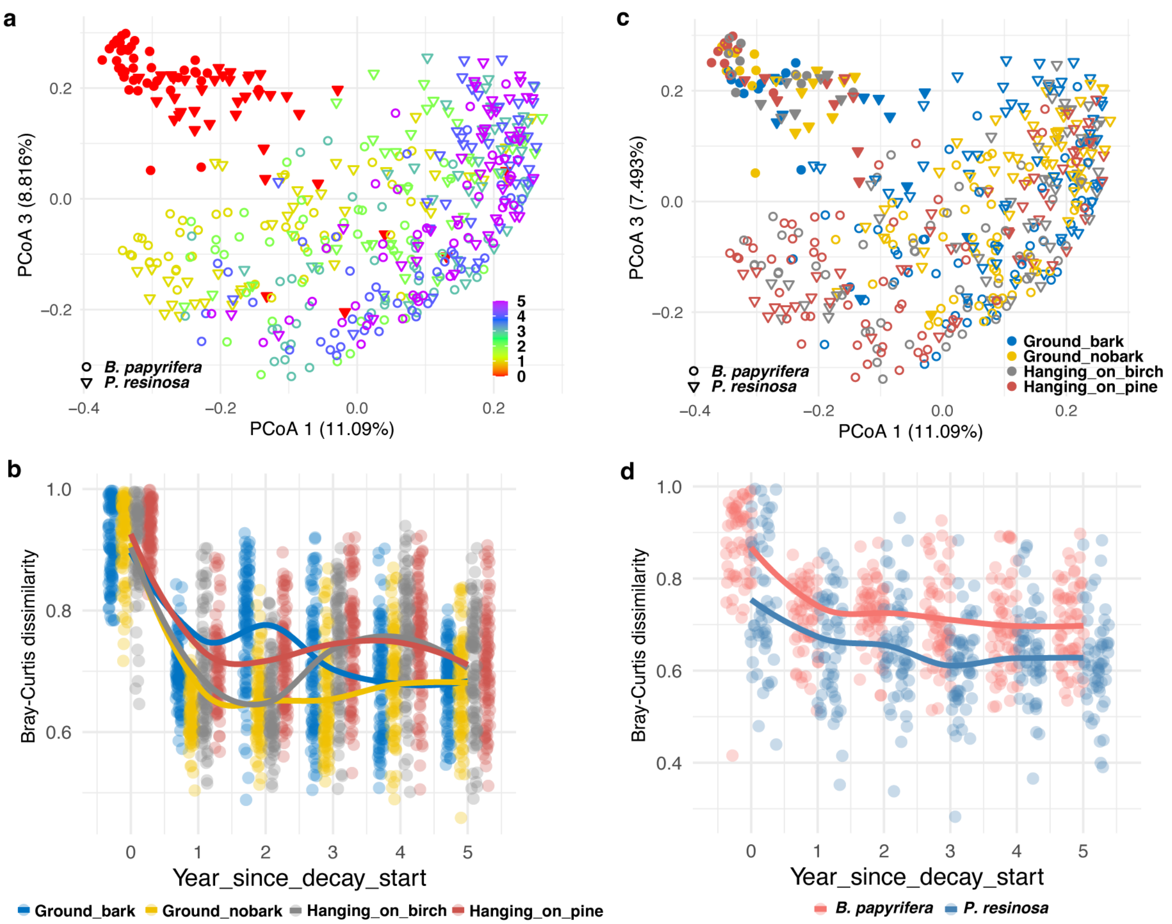


**Fig. S13 The structure of bacterial community decaying *B. papyrifera* and *P. resinosa* in different treatments over 5 years.** (**a**) PCoA of Bray-Curtis dissimilarity of bacterial communities between samples shaped by tree host and colored by time, while sound and decayed wood are represented by filled and open symbols, respectively. (**b**) Pairwise distances between *B. papyrifera* and *P. resinosa* within each decay time and each treatment. (**c**) PCoA of bacterial communities shaped by tree host and colored by treatments. The sound and decayed wood samples are represented by filled and open symbols, respectively. (**d**) Pairwise distances between treatments within each decay time and each tree host.

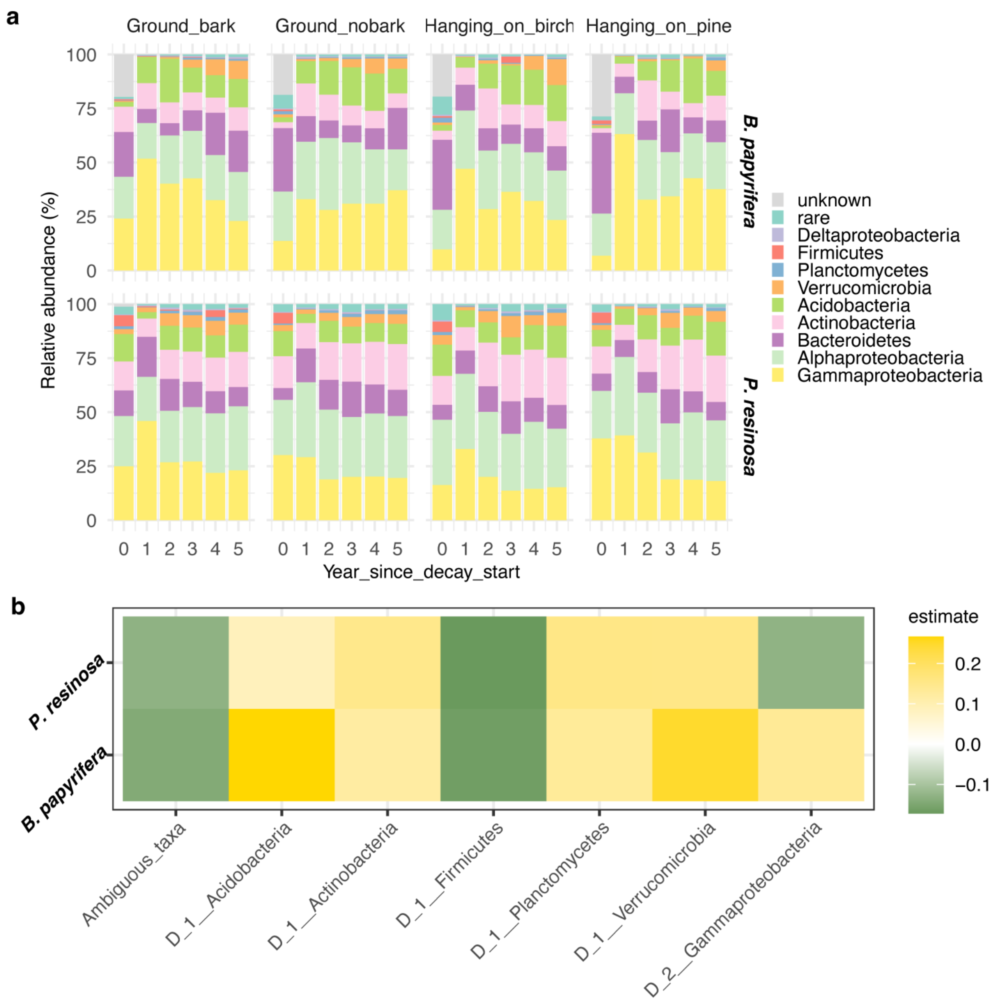


**Fig. S14 Bacterial community composition in different decay treatments over 5 years.** (**a**) Relative abundance of bacterial communities in the decay process of *B. papyrifera* and *P. resinosa* at the phylum level. The *Proteobacteria* phylum was divided into its respective class levels, as they made up a large portion of our deadwood bacterial communities. (**b**) Beta regression coefficient estimates for phyla that are increasing (above 0) or decreasing (below 0) in relative abundance over decay time. Beta regression was performed using the "BetaReg" package in R.

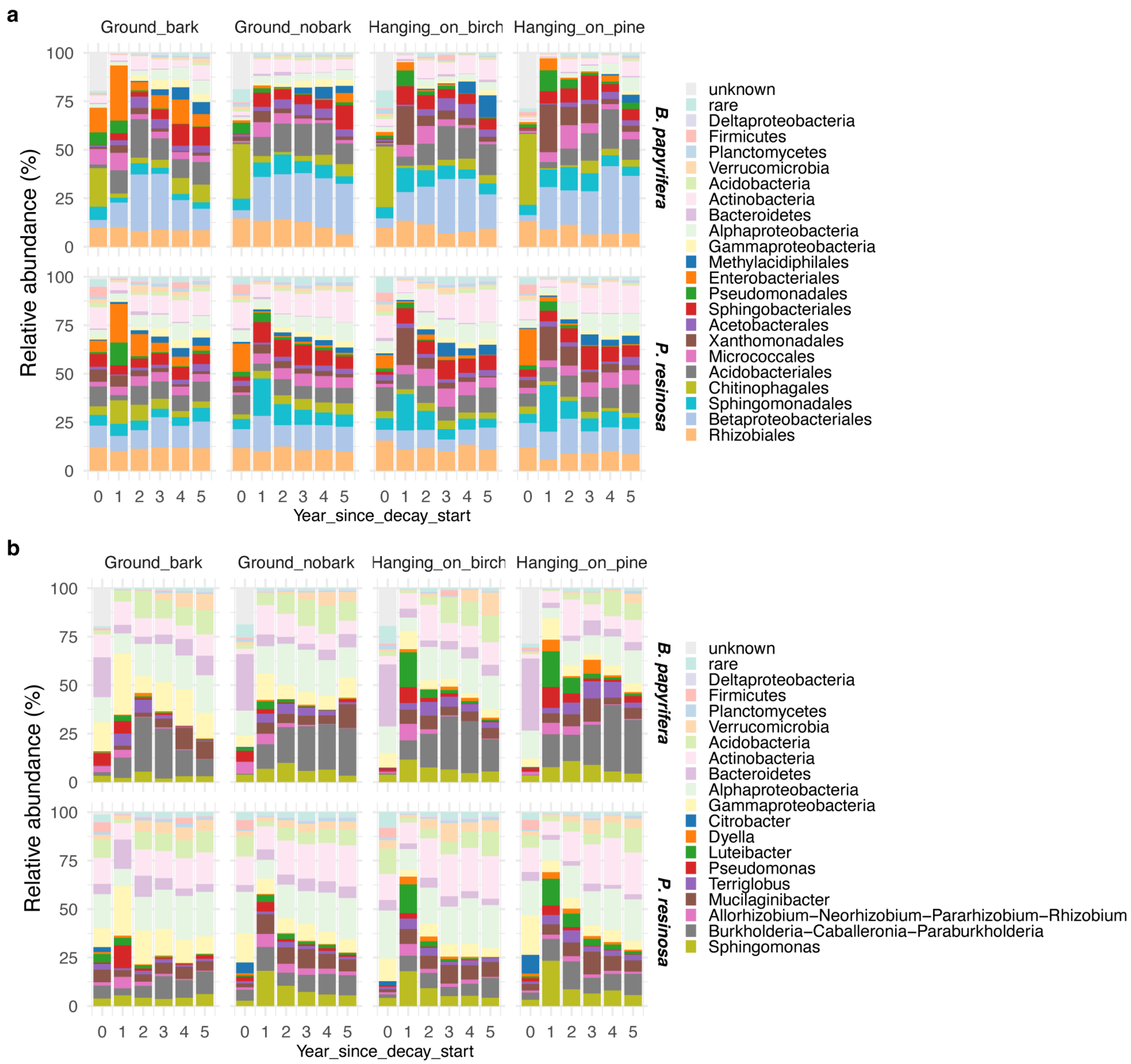


**Fig. S15 Relative abundances of bacterial taxa in the decay process of *B. papyrifera* and *P. resinosa* at the order level (a) and genus level (b) for different treatments.** Only orders and genera with at least 7% relative abundance in one of the treatments were specified, and all others were classified as the rare group. All the taxa not assigned to the level of order or genus were classified as the unknown group.

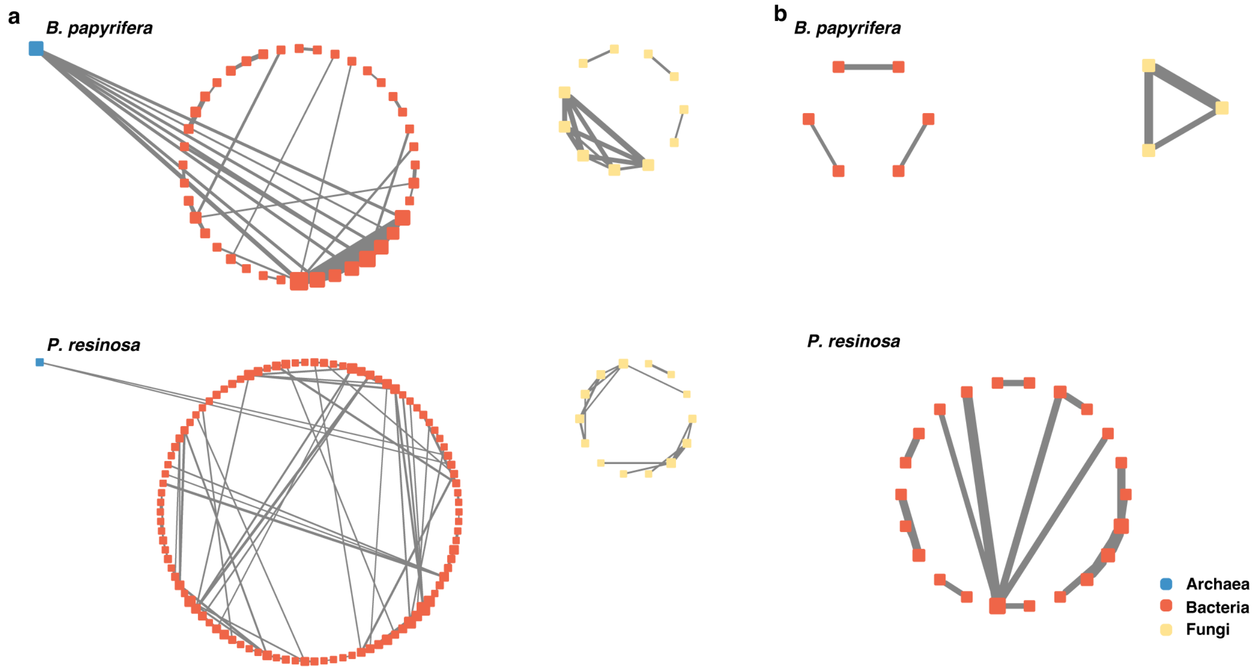


**Fig. S16 Network co-occurrence analysis of fungal and bacterial community for *B. papyrifera* and *P. resinosa* at the OTU level (a) and genus level (b).** Connections represent Spearman's correlations with adjusted *P* values (correlation estimate < −0.8 or > 0.8 and Benjamini-Hochberg adjusted $P < 0.01$). The size of the node corresponds to the number of connections (degree). The width of the edge corresponds to the value of correlations (weight).
